# Differential effects on membrane permeability and viability of human keratinocyte cells undergoing very low intensity megasonic fields

**DOI:** 10.1038/s41598-017-16708-4

**Published:** 2017-11-28

**Authors:** F. Domenici, F. Brasili, S. Giantulli, B. Cerroni, A. Bedini, C. Giliberti, R. Palomba, I. Silvestri, S. Morrone, G. Paradossi, M. Mattei, F. Bordi

**Affiliations:** 1 Dipartimento di Scienze e Tecnologie Chimiche, Università degli Studi di Roma “Tor Vergata”, Rome, Italy; 2 Dipartimento di Fisica, Università degli Studi di Roma “Sapienza”, Rome, Italy; 3 Dipartimento Innovazioni Tecnologiche e Sicurezza degli Impianti, Prodotti e Insediamenti Antropici (DIT), INAIL, Monteporzio Catone, Rome, Italy; 4 Dipartimento di Medicina Molecolare, Università degli Studi di Roma “Sapienza”, Rome, Italy; 5 Dipartimento di Medicina Sperimentale, Università degli Studi di Roma “Sapienza”, Rome, Italy; 6 Centro Servizi Interdipartimentale - Stazione Tecnologia Animale and Dipartimento di Biologia, Università degli Studi di Roma “Tor Vergata”, Rome, Italy; 7 Istituto dei Sistemi Complessi, Consiglio Nazionale delle Ricerche, Florence, Italy

## Abstract

Among different therapeutic applications of Ultrasound (US), transient membrane sonoporation (SP) - a temporary, non-lethal porosity, mechanically induced in cell membranes through US exposure - represents a compelling opportunity towards an efficient and safe drug delivery. Nevertheless, progresses in this field have been limited by an insufficient understanding of the potential cytotoxic effects of US related to the failure of the cellular repair and to the possible activation of inflammatory pathway. In this framework we studied the *in vitro* effects of very low-intensity US on a human keratinocyte cell line, which represents an ideal model system of skin protective barrier cells which are the first to be involved during medical US treatments. Bioeffects linked to US application at 1 MHz varying the exposure parameters were investigated by fluorescence microscopy and fluorescence activated cell sorting. Our results indicate that keratinocytes undergoing low US doses can uptake drug model molecules with size and efficiency which depend on exposure parameters. According to sub-cavitation SP models, we have identified the range of doses triggering transient membrane SP, actually with negligible biological damage. By increasing US doses we observed a reduced cells viability and an inflammatory gene overexpression enlightening novel healthy relevant strategies.

## Introduction

Over the last few years, Ultrasounds (US) have gained an increasing interest among the scientific community for their use in diagnostic imaging and, also due to the design of novel multitasking contrast agents^1–3^, for the compelling opportunities they offer to improve several emerging therapeutic applications, like gene and drug delivery^4–7^. It is thought that US propagation through the different layers of the skin and the underlying tissues, by transferring mechanical energy towards specific sites of the body, induce a temporary, non-lethal porosity in cell membranes^8^. This phenomenon, known as transient membrane sonoporation (SP), could lead to the concrete development of new therapeutic approaches towards a more efficient and safer release of cargo drugs *in situ* ^9^. In this respect, SP has been shown to facilitate the uptake of low- and high-molecular-weight molecules and has been effectively exploited to obtain high drug concentrations in specific sites of interest, in order to minimise systemic side effects^4,10–12^.

Despite the widespread use of US in diverse application fields, the sensitivity of tissues and biological membranes to the induced mechanical stress is not fully understood^13^. In this regard, the US-induced cytotoxic effects, related to the failure of the cell repair mechanisms and to the possible activation of inflammatory pathways could represent a serious risk to human health^10,11^. Moreover, exposure to US could weaken the tight packing arrangement between the skin cells, thus hindering their role as protective barrier of the underlying tissues. This phenomenon, known as sonophoresis, has been widely employed for enhanced transdermal drug delivery^4,14,15^, and also raises new possibilities for delivering therapeutic agents directly into the brain through the human Blood Brain Barrier (BBB), in a non-invasive, localized and transient manner^6,16,17^. However it could also facilitate the access of different exogenous particles, such as metal, organic and biological (e.g. viruses, bacteria or parasites) corpuscles^13,18^.

Most of the damages caused by US exposure occur when their energy exceeds the so called cavitation threshold (i.e. for spatial peak temporal average intensity I_spta_ > 100 mW/cm^2^). Above this threshold, oscillating pressure fields produce micrometer and sub-micrometer gas bubbles^19,20^ which, oscillating and collapsing, could induce in cells and tissues severe biological effects^19,20^. However, biological alterations related to the membrane SP have also been detected below the cavitation threshold^13,21–24^. Recently, a unified elastic model, named BiLayer Sonophore (BLS), has been proposed to explain the biological effects of US exposure in both cavitation and sub-cavitation regimes^24^. The model, supported by simulations and experiments, shows that as the mechanical energy transported by US is absorbed by the membrane, the intermembrane space between the bilayer leaflets starts to expand and contract, despite the molecular attraction forces. This effect strongly enhances the permeability of the membrane, facilitating the uptake of drugs and genetic material^13^.

Spectroscopic instrumental methods allow for sensitive detection of structural and functional changes in cells associated with US exposure. These methods demonstrated very effective to study bioeffects of US in the cavitation regime^21^ and can be suitably employed to investigate the sub-cavitation one, whose complete characterisation is still lacking. Using these methods, we pointed out in previous studies that low-intensity 1 MHz US enhances the membrane permeability of murine immortalised fibroblasts NIH-3T3, and might also induce potential cytotoxic and genotoxic effects^13,22^. This cell line is often used as a model to analyse alterations in plasma membrane properties and viability of connective tissue, one of the skin components.

Here we investigate the *in vitro* effects of very low-intensity 1 MHz US in human keratinocytes (HaCaT) cell line. This spontaneously immortalized cell line is a widely employed model of keratinocytes cells composing the epidermal barrier which protects humans against the environmental damage. It is due to its ease of propagation and being nearly normal phenotype. As well known these cells are also able to produce cytokines involved in inflammatory responses and cell death. We focus on keratinocytes because in the majority of non invasive medical treatments 1 MHz US interacts first with these cells (e.g. either to facilitate subcutaneous permeation of drugs or to operate non-invasive treatments into a deeper area of the body).

We reported herein microfluorescence and flow cytometry combined experiments at varying time and intensity of US exposure, aimed to evaluate the alteration induced in membrane permeability and viability of HaCaT cells. Since under equal exposure conditions the US bioeffects may be dependent on cell-line type, comparative experiments were undertaken on HaCaT and NIH-3T3 cell lines. The US intensity dependence on the molecular weight (MW) of the internalised molecule is also discussed. Concomitant effects on cell viability are shown and discerned in terms of necrosis and apoptosis using AnnexinV-APC (allophycocyanin)/7AAD (7-amino- actinomycin) and Propide Iodide (PI) combined assays.

Finally, we focused on the possible activation of an inflammation pathway which has been intensively investigated in the last few years to identify the mechanisms that link chronic inflammation to neoplastic progression and metastasis^25,26^. As already mentioned, US shows a wide versatility in several therapeutic applications. Indeed, in recent years, the therapeutic benefits provided by US in inflammation relief have been much studied^26^. In the light of these evidences, we analysed by polymerase chain reaction (PCR) the gene expression of the inflammatory marker Interleukin 6 (IL-6).

Our results point out that even very low-intensity US (i.e. well-below intensity of bubble cavitation generation) significantly enhance the membrane permeability of HaCaT cells through SP, according to BLS model^24^. The corresponding cytotoxic and inflammatory responses were also characterised and threshold exposure parameters for their activation were identified. Notably, it was possible to identify a range of treatment doses in which the SP is activated with negligible toxicological effects.

## Materials and Methods

### Cell and culture conditions

All experiments were carried out using the human keratinocyte cell line HaCaT. Comparative sonoporation experiments (see section 3.1) were performed using the murine fibroblast cell line NIH-3T3. The cells were grown in Dulbecco’s modified Eagle’s medium (DMEM, Sigma-Aldrich, St. Louis, MO). All media were supplemented with 10% fetal bovine serum (Euroclone), 2 mM glutamine, 100 U penicillin/100 μg streptomycin per ml. Cells were maintained in a tissue culture incubator at 37 °C, 5% CO_2_. Cells were seeded in 9.6 cm^2^ cell culture-treated Petri dish (Falcon^®^ Easy Grip^TM^, Becton Dickinson Labware, Franklin Lakes, NJ; © ibidi GmbH, Martinsried, Germany) at 7–9 × 10^5^ cells/ml. In each experiment culture media was replaced with 3 ml of Dulbecco’s Formula Phosphate Buffered Saline without calcium and magnesium (PBS, Sigma- Aldrich, St. Louis, MO).

### Ultrasound exposure system and experimental protocol

The setup for the US exposures was a homemade device consisting of a signal generator (Agilent 33220 A), a signal amplifier (Amplifier Research 25A250) and a submersible piezo-ceramic circular transducer (44 mm diameter) tuned at 1 MHz (Precision Acoustics Ltd) (for details see also Section 1 of ESI). Such a system provided well defined sinusoidal US waves independently from the exposure conditions and with different amplitude (see below), both in continuous and pulsed mode. To assess the relevance of our study, the exposure conditions were compared with those actually employed in medical treatments by using a second device (Nuova Elettronica, Italy) consisting of a submersible piezo-ceramic circular transducer (60 mm diameter) tuned at 1 MHz, and its generator.

The exposure setup was made of a tank (30 × 30 × 30 cm) filled with degassed Milli-Q water (18.2 MΩ·cm, resistivity) with the transducer placed at the bottom. Acoustic absorbers were employed in order to suppress reflected waves from the tank. The temperature of the water bath was kept constant at 25 °C. A hermetically sealed cell culture-treated polystyrene Petri dish (~9.6 cm^2^) containing 3 ml of PBS solution was positioned above the transducer at the water surface, submerged up to half of its thickness (aligned to the water-air interface of the external water tank). The Petri thicknesses were ~0.90 mm (Falcon) or ~0.17 mm (ibidi), the latter allowing the use of high-resolution immersion objectives.

The acoustic fields were characterised measuring their intensity transmitted from the Petri dish bottom area (where the cells are located) with a needle hydrophone of 0.5 mm diameter (Precision Acoustics, UK) with sensitivity 483 mV/MPa at 1 MHz endowed with a micrometric positioning system. By calibration we can measure the US pressure and, ultimately, the intensity of the ultrasound that the cells onto the Petri actually undergo.

In this work, the US intensities employed are provided in terms of Spatial Peak Temporal Average SPTA (I_spta_), i.e. the maximum spatial intensity measured when the pulse is activated, averaged for the period of pulse repetition. This quantity is commonly used in the characterisation of the biological effects induced by low intensity US. In all the experimental conditions employed the I_spta_ values (varying from 7 to 16 mW/cm^2^) were well below 100 mW/cm^2^ which is for general consensus the cavitation threshold. US exposure time of 5′, 15′, 30′ and 45′ were chosen.

According to therapeutic protocols, the samples were sonicated at constant Source-dish Surface Distance (SSD), i.e. keeping constant their distance from the transducer. The SSD was fixed at 7 cm, to minimise close-field effects. Geometries of Petri and of overall exposure setup space pattern were set in order to avoid matching with the resonance length of the US stimulus. We checked the results with or without pulse repetition frequency sets, checked the sinusoidal waveform with respect to that of the US source to exclude multiple harmonic generation. The temperature of water and of the Petri remains stable within 1 °C also under US continues sinusoidal regime after 1 hour exposition, as measured by digital thermocouple (0.1 °C, accuracy).

According to the literature^27^ those exposure conditions were chosen to exclude or render ineffective perturbations which can affect reproducibility of calibration and generate *in vitro* response artefacts. Variability of the I_spta_ up to 20 mW/cm^2^ was within 10% thus providing a stable and reproducible US field calibration. The acoustic attenuation due to the Petri dish surface was below 4% depending on the Petri thickness.

### Optical fluorescence microscopy

For fluorescence measurements an inverted optical microscope Leica DMIL (Obj 10×; 20×; 40×; 100×) equipped with Hg vapour lamp and optical filters DAPI/FITC/TRITC (FITC: BP 450–490 nm excitation, LP 515 nm emission; TRITC: BP 515–560 nm excitation, LP 590 nm emission) and ZEISS AxioCam ICc3 video camera was used. The sample consists of HaCaT cell cultures incubated in Petri dishes until subconfluence. Immediately before to US application, in each sample the culture medium was replaced by a solution of the green fluorescent dye calcein (Molecular Probes, Eugene, OR, MW = 622.54 Da, wavelengths of the maximum absorption and emission peaks at 494 nm and 514 nm, respectively) in PBS, with a final concentration of 10 µM^28^. Calcein is not able to permeate cell membrane, therefore the analysis of its uptake allowed us to evaluate the formation of sonopores^29^. Immediately after sonication, a recovery time was scheduled for the resealing of the membrane pores in viable cells; based on preliminary experimental study, a recovery of 10′ is sufficient to this aim. Afterwards, cells were washed with PBS to remove the non-internalized calcein. Moreover, 20 µl of red fluorescent Propidium Iodide (Sigma-Aldrich, St. Louis, MO, MW = 668.4 Da, wavelengths of maximum absorption and emission peaks at 535 nm and 617 nm, respectively) was added before the microscopy analysis to estimate non-viable cells. In fact, PI can cross only the damaged membranes, and intercalate in the cellular DNA, staining the non-viable cells and emitting fluorescence.

In addition, the SP effects induced by US were studied using the fluoroprobes FITC-dextrans (Sigma-Aldrich, St. Louis, MO) with different MW (10, 40 and 70 kDa), to investigate the size of the induced sonopores depending on I_spta_ exposure^30^.

### Confocal laser scanning microscopy

A Nikon Eclipse (Ti-E) inverted C1 confocal microscope (Obj 20×; 40×; 60×; 100×) equipped with two lasers and a motorized stage was used. The lasers mounted on the microscope are: an Argon ion (Spectra Physics, Mountain View, California) laser emitting fluorescence at 488 nm wavelength and He-Ne laser (Melles Griot Florence, Italy) emitting fluorescence at 543 nm wavelength.

For scanning along the axial (z) dimensions, a Plan Apo 60×, high numerical aperture (NA = 1.40) oil immersion objective (Nikon Florence, Italy) was chosen and a Z-stack of images was acquired at 0.15 μm increments, according to Nyquist criterion, automatically calculated by the Nikon EZ-C1 acquisition software. For 3D image reconstruction, the NIS Elements AR 4.30 software (Nikon Florence, Italy) was used.

### Flow cytometry analyses

Fluorescence activated cell sorting was performed using flow cytometer FACSCalibur (BD Biosciences, Singapore) equipped with two excitation lasers (Argon ion and visible red diode lasers at 488 nm and 635 nm wavelengths, respectively), fluorescence channels detect light at 530 ± 15 nm (FL1, Calcein), 585 ± 21 nm (FL2, PI); for each sample 10,000 events were recorded. Preparation and treatment of cells were performed according to the fluorescence microscopy procedure. Before each analysis, the fluoroprobe calcein was added to the culture medium to evaluate the uptake efficiency after US exposure. Results were expressed in percentage of positive cells (% gated), using the CellQuest software (Becton Dickinson).

Furthermore, flow cytometry analysis was performed to evaluate apoptosis versus necrosis using AnnexinV-APC/7AAD in a calcium binding buffer (BD Biosciences kit) and PI combined assay, according to the manufacturer’s protocol.

### Reverse transcriptase polymerase chain reaction (RT-PCR) assay

Total mRNA from HaCaT was extracted using Trizol reagent (Euroclone, Pero, MI) according to the manufacturer’s instructions. High quality RNA preparation was re-suspended in nuclease-free Milli-Q water (18.2 MΩ·cm, resistivity) water and subjected to semi-quantitative polymerase chain reaction; Moloney murine leukemia virus (M-MULV, New England BioLabs, UK) and reverse transcriptase was used to convert 1 µg of total mRNA into cDNA at 42 °C. 5 µg of each cDNA was then subjected to RT-PCR in a buffer containing 25 pmol of upstream and downstream primers (Sigma-Aldrich, St. Louis, MO) and 1.25 U of Platinum Taq polymerase (New England BioLabs, UK). The sequences of forward and reverse, respectively, human gene-specific primers, the conditions of amplification as well as the amplified products size are the following: glyceraldehyde-3-phosphate dehydrogenase (GAPDH): 5′-ACATGTTCCAATATGATTCC-3′, 5′-TGGACTCCACGACGTACTCAG-3′ 60 °C ×30″180 bp; IL-6: 5′-CCTCCAGAACAGATTTGAGA-3′, 5′-CCTTAAAGCTGCGCAGAATG-3′ 56 °C ×1′ 280 bp.

The amplification reaction was carried out in Piko-Thermal Cycler (Finnzymes Instrument). The thermal profile for PCR amplification was as follows: GAPDH, 94 °C for 30″, 60 °C for 30″, 72 °C for 30″ for a total of 30 cycles; IL-6 (right), 95 °C for 50″, 56 °C for 1′, 73 °C for 15″ for a total of 30 cycles. The amount of amplified products, expressed in arbitrary optical density units, was normalized with GAPDH as housekeeping gene. The resulting PCR products were separated in 2% agarose gel and visualized with Gel-Red (GelRed nucleic acid gel stain, Biotium, Hayward, CA).

## Results and Discussion

The effect of the exposure to very low intensity US (I_spta_ between 7 and 16 mW/cm^2^, well below the cavitation threshold, 100 mW/cm^2^) with a frequency of 1 MHz was studied on HaCaT cells. The experiments were performed with an equivalent medical setup sketched in Figure S1 of ESI, at varying treatment parameters, such as I_spta_, duty cycle, and sonication time. Preliminary experiments pointed out that, in the range of exposure parameters involved herein, the temperature remained constant (within 1 °C) excluding the thermal dissipation of the US energy. Moreover, the treatments did not show any significant dependence of the studied effects on the US wave duty cycle. These observations are consistent with the low US absorption at 1 MHz; on the contrary, thermal effects and duty cycle could affect cells and tissues exposed to higher frequencies, being the US attenuation coefficient proportional to the frequency^23,31^.

Results will be initially presented considering the two relevant exposure parameters, sonication time and I_spta_, separately. The structural and physical alterations of the cell membrane (permeability, uptake efficiency and maximum size of the internalized molecules) will be analysed and correlated to the corresponding cytotoxic damage and to the possible activation of inflammatory patterns. Afterwards, the results will be interpreted in terms of the sonication dose, defined as the product of the I_spta_ by the exposure time, namely the surface density of the acoustic energy incident on the sample during sonication. Actually, the studied phenomena depend on this quantity and this allowed us to identify a range of exposure parameters where a transient SP occurs with negligible biological damages.

### Analysis of the influence of US exposure time

Alterations in the membrane permeability of HaCaT cells induced by low-intensity 1 MHz US were estimated in terms of the uptake efficiency of the green fluorescent probe calcein. Since intact membranes are impermeable to calcein, it represents a reliable marker for SP. The percentage of green-positive cells was quantified by flow cytometry analyses according to Section 2.5.

**Figure 1:**
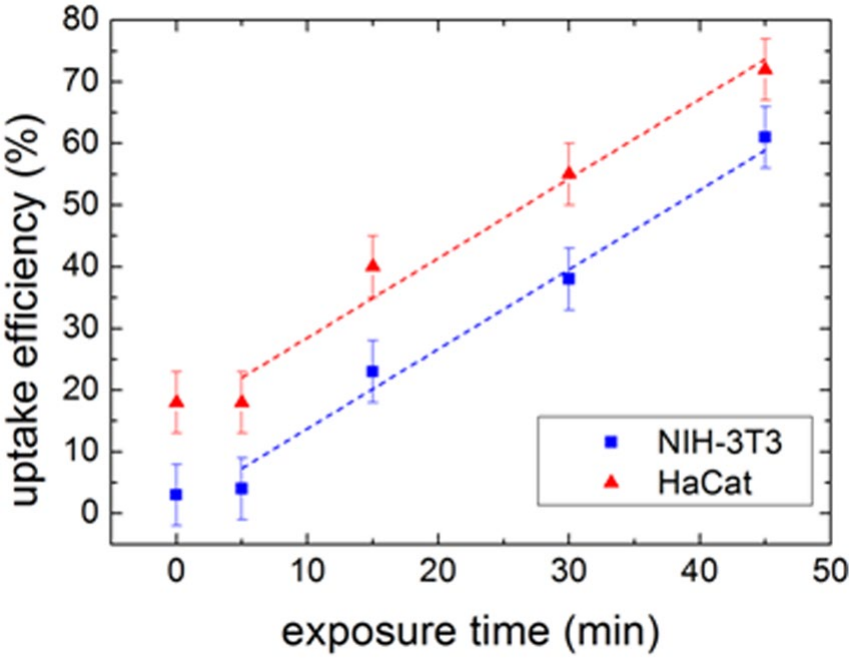
Uptake efficiency versus exposure time, evaluated by flow cytometry analysis on NIH-3T3 and HaCaT cell lines treated with 1 MHz US at I_spta_ = 7 mW/cm^2^. Dashed line: linear regression fits (correlation coefficient R = 0.993, p = 0.00075 blue line; R = 0.986, p = 0.0144 red line).

In order to evaluate the sensitivity of membrane permeability to US exposure time, a very low US I_spta_ was used. Notably, already a I_spta_ of 7 ± 1 mW/cm^2^ significant effects on membrane permeability can be triggered by proper exposure times. Representative results of parallel flow cytometry investigation on HaCaT and NIH-3T3 cells both sonicated at I_spta_ ~7 mW/cm^2^ for 5′, 15′, 30′ and 45′ are shown in Fig. 1 (red triangles and blu squares, respectively). As expected, the comparison between cell lines after low sonication times (below the threshold of sonoporation) results in a little affinity of calcein, where the differences (the highest in HaCaT cells) should account for their respective membrane compositional and permeability features. More important, both cell lines show a significant increase of the uptake efficiency starting from 15′ of exposure, which corresponds to a sonication dose of (6.3 ± 0.9) J/cm^2^, suggesting the activation of the SP process. According to the best fits of Fig. 1, for longer exposure times, our experiments point out a linear correlation between uptake efficiency and exposure time, with an identical uptake rate induced by US in both HaCaT and NIH-3T3 cells (linear regression slopes (%/min) 1.3 ± 0.2, and 1.4 ± 0.2, for HaCaT and NIH-3T3, respectively). This would mean that the uptake efficiency increase due to SP is a cell-line-independent phenomenon.

**Figure 2:**
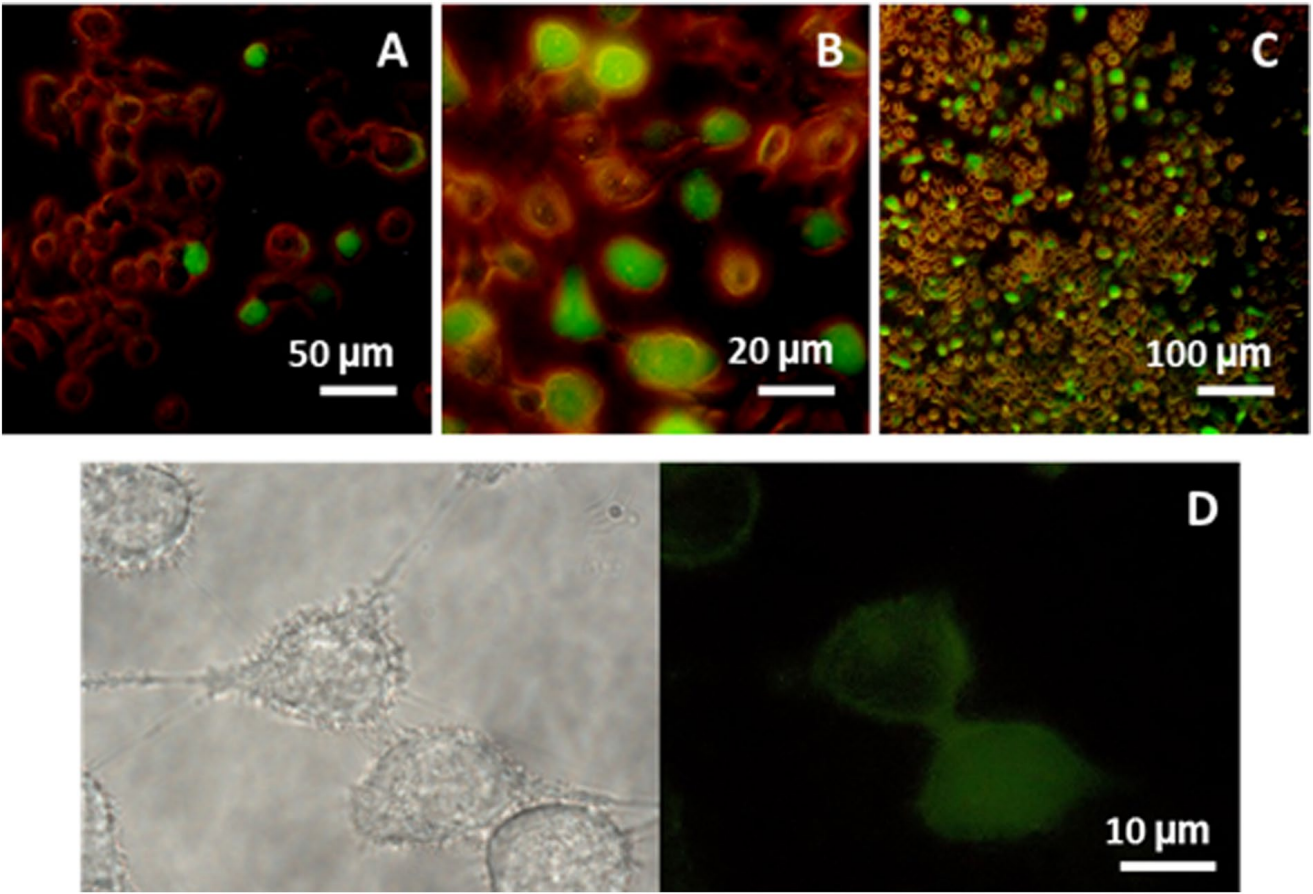
Microscopy images acquired on HaCaT cells treated with 1 MHz US at I_spta_ = 7 mW/cm^2^. FITC fluorescence together with low intensity transmitted phase contrast images of cells sonicated for 15′ (**A**), 30′ (**B**) and 45′ (**C**); detail of cells sonicated for 45′(**D**), acquired in phase contrast (left) and fluorescence (right), where a two-level uptake of the fluoroprobe (diffused in the cytosol and localized in the biological membrane) is evident.

**Figure 3:**
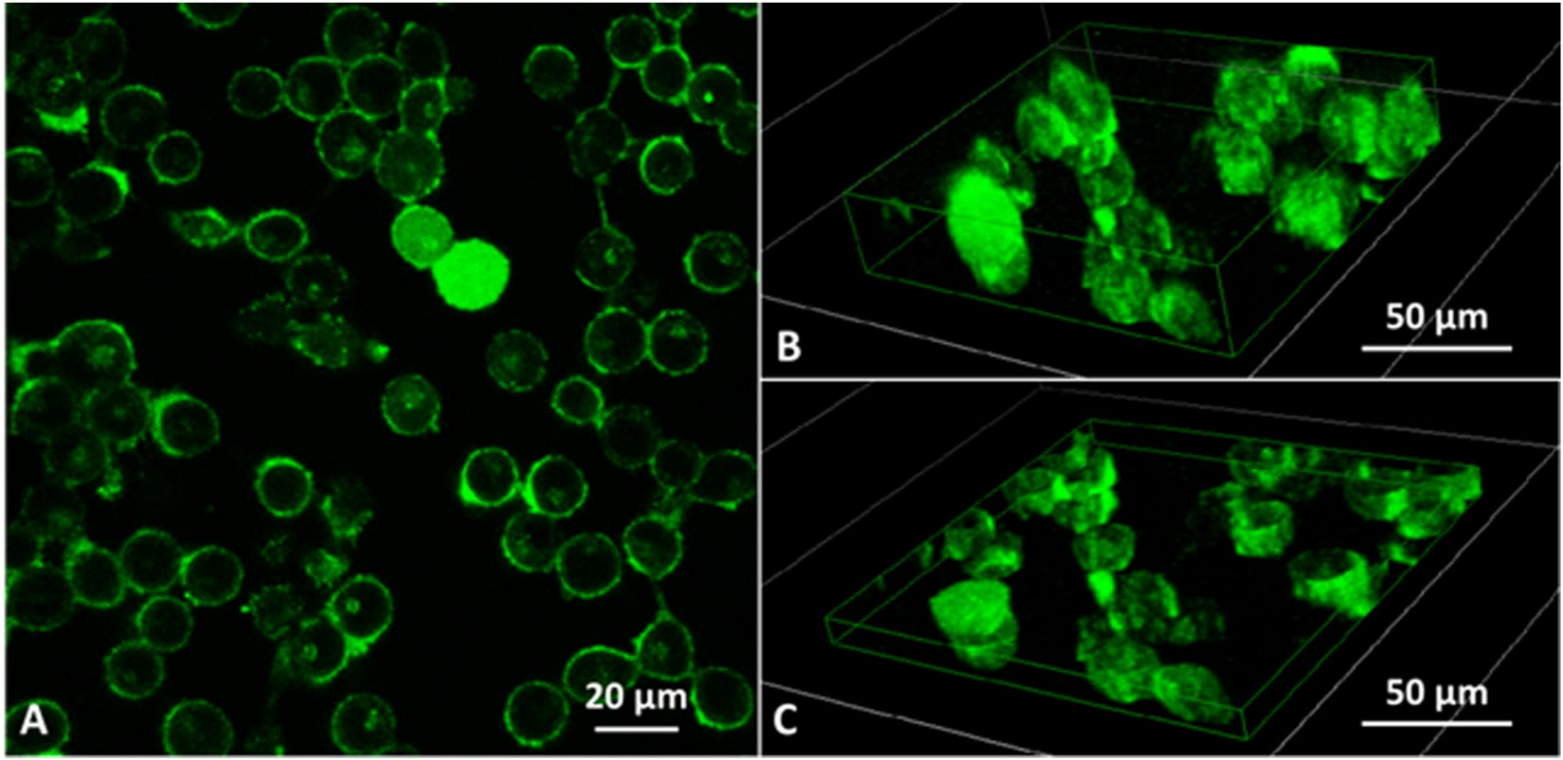
(**A**) Representative image acquired on HaCaT cells treated for 30′ with 1 MHz US at I_spta_ = 7 mW/cm^2^ with a confocal fluorescence microscopy; (**B**) and (**C**) 3D reconstruction of cells that internalised the fluorescent dye calcein: the section (**C**) evidences a two level uptake, diffused in the cytosol and localised on the biological membrane.

To further investigate the SP process and characterise the internalization, treated HaCaT cells were evaluated by fluorescence microscopy. Consistent with flow cytometry results, samples sonicated for 5′ do not show any significant alteration in the membrane permeability with respect to the control (untreated) sample, while starting from 15′ an increasing uptake efficiency is observed (as shown in Fig. 2 and Figure S2 of ESI) although not all cells showed fluorescence. Correspondingly, cells appear less dense and adherent with respect to the control sample, suggesting a loosening of tight-junctions. The representative images of Fig. 2 suggest a heterogeneous localization of the calcein within the cells, and in fact confocal fluorescence microscopy allowed to clearly distinguish within HaCaT cells the peripheral distribution of the probe from the internal one (see image and 3D reconstructions of Fig. 3). Further detailed images are shown in Section 3 of ESI. Observations are consistent with a two-levels uptake of the fluoroprobe: a peripheral one, where the probe is localized in the endoplasmic reticulum network around the plasma membrane; the other one, exhibited significantly after 30′ exposure, where the probe diffusion into the cytoplasm causes an intense fluorescence uniformly distributed throughout the whole cell, including nucleoplasm. It should be noted that Stokes diameter of calcein is ~1.36 nm and its intranuclear transport is not hindered by diffusive barriers^29^. A more complete analysis reported in Sections 3.2 and 3.3 allows to conclude that the US dose of exposure and the molecular weight of the probe were dominant factors in determining the uptake.

**Figure 4:**
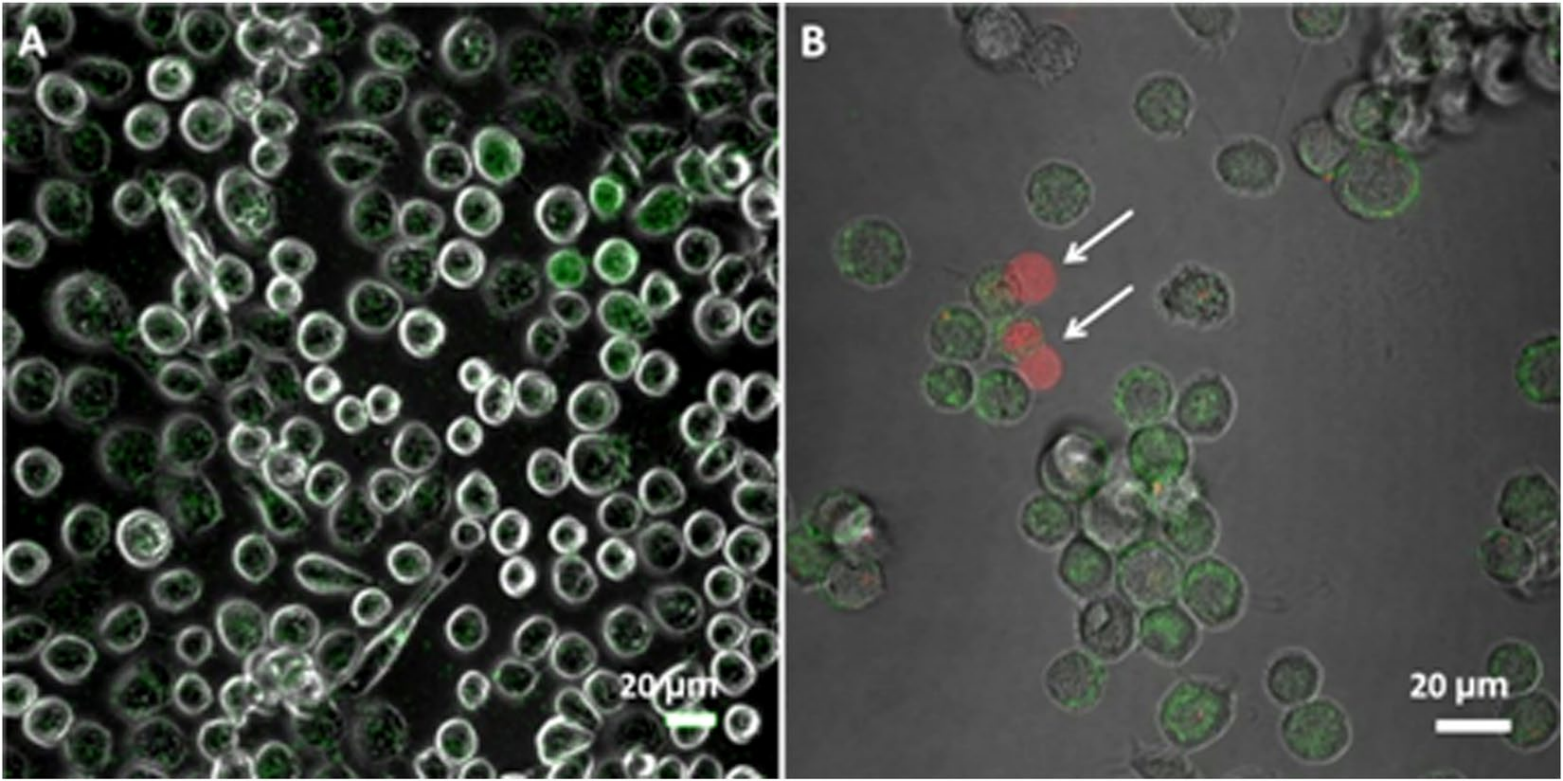
Confocal fluorescence (calcein, PI) merged with low intensity transmitted phase contrast images acquired on HaCaT cells treated for 30′ (**A**) and 45′ (**B**) with 1 MHz US at I_spta_ = 7 mW/cm^2^; in the second image (**B**) white arrows indicate the colocalization of calcein and PI in the cells, pointing out the failure of the recovery process after the SP.

A further assay based on the red PI fluoroprobe was performed to evaluate the recovery of the native membrane permeability of sonicated cells (see also Section 2.3). The internalization of PI denotes the activation of cytotoxic processes. The co-localization of the green and red dyes inside the cells indicates therefore the failure of the membrane recovery process after SP. On the other hand, the observation in cells of the green fluorescence in the absence of the red one points out the occurrence of membrane SP during the US treatment and its successful recovery after the field switching off. Representative confocal fluorescence images are shown in Fig. 4. The co-localization of the two dyes is absent in the cells treated for 15′, while it is scarcely observed after 30′ of exposure (Figure S5 of ESI). Consistently, a net loss of viability of ~5% was revealed, as checked by flow cytometry. In the cells sonicated for 45′, corresponding to a dose of (19 ± 3) J/cm^2^, the co-localization appears more evident (see white arrows in Fig. 4B) and a more significant loss of viability occurs (~10%, as checked by flow cytometry). These findings suggest that a longer exposure time, corresponding to a higher dose at fixed I_spta_, induces an irreversible alteration in the membrane structure followed by a consequent cytotoxic response.

### Analysis of the influence of US intensity

**Figure 5:**
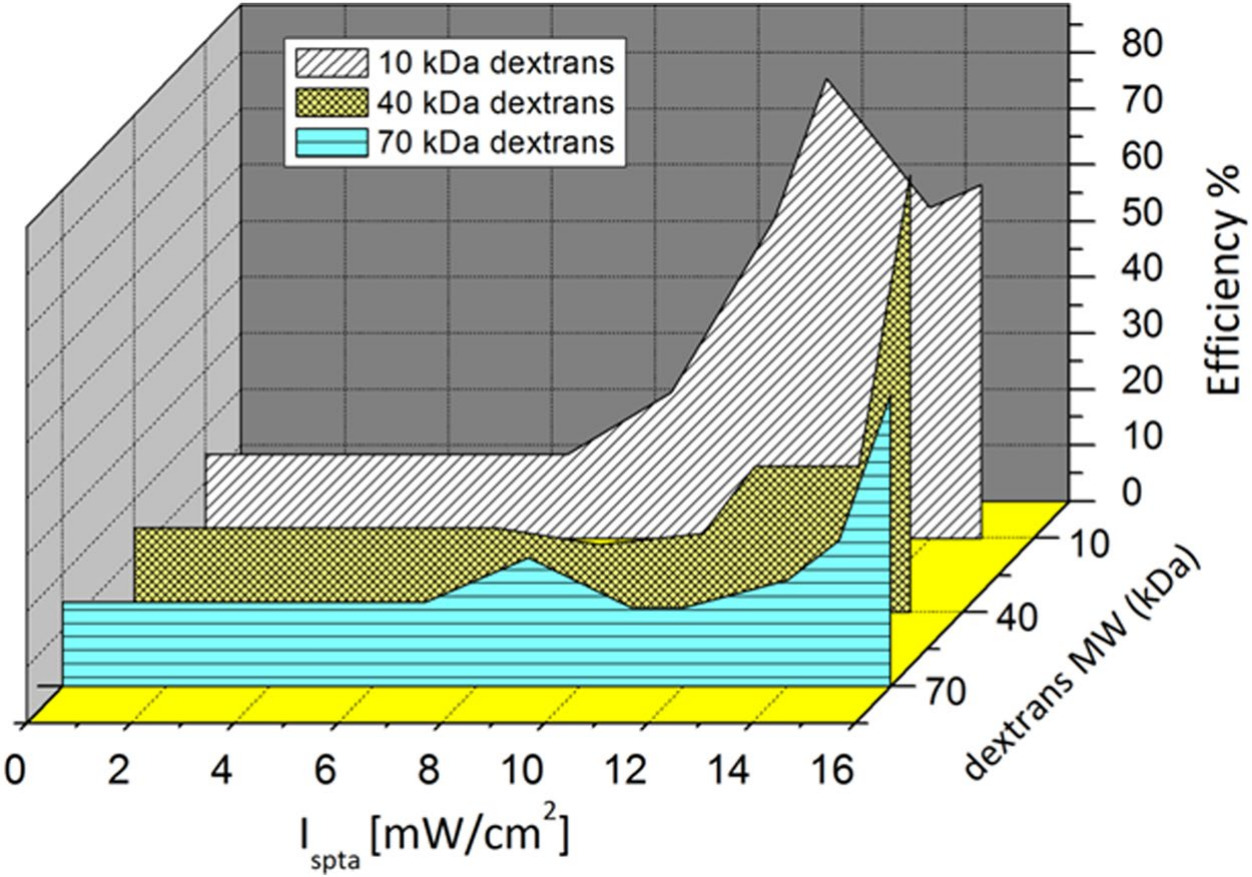
Uptake efficiency of FITC-labeled dextrans with different MWs, evaluated by flow cytometry at varying exposure I_spta_ on HaCaT cells sonicated for 15′ at 1 MHz.

The effect of varying the intensity of applied US field on the membrane permeability was analysed on HaCaT cell line after 15′ of exposure, short enough to exclude effects induced by a long-lasting sonication. In our study US intensities were in the range between I_spta_ = (7 ± 1) mW/cm^2^ and I_spta_ = (16 ± 1) mW/cm^2^, well below the cavitation threshold^24^. According to the protocol described in Sections 2.3 and 2.5, FITC-labelled dextrans of different MW (MW = 10, 40 and 70 kDa) were used to characterise, for each US intensity, the induction of membrane permeability to the different molecule size. This procedure allows in turn to evaluate the size of the sonopore. Similarly to calcein, dextrans do not enter significantly the untreated cells^31,32^, while in the cavitation US regime the cell uptake of FITC-labelled dextrans can be increased strongly by both SP and by promotion of endosome trafficking^30^. In this regard, the results of flow cytometry measurements reported in Fig. 5 clearly show a size-dependent uptake, a phenomenon which allows us to exclude the concomitance of active endocytosis effects and, according to the literature^22^, suggests the low intensity-triggered SP as the main mechanism involved in the transport of dextran molecules through the plasma membrane. Specifically, the observed SP-induced uptake is characterised by a threshold I_spta_, linearly increasing with the increase of the MW of the internalised molecules. Below this threshold the uptake efficiency of treated cells is comparable to that of nonexposed ones. The threshold I_spta_ values are reported in Table 1. For intensities above the threshold, the uptake efficiency increases with the increase of I_spta_ until a maximum is reached. For the 10 kDa dextran, then it slightly decreases to a plateau. The observed decrease of the uptake efficiency at high intensities can be explained by the increase of the number of dead cells, which are removed from the sample when rinsing, thus not contributing to the flow cytometry analyses. Furthermore, the activation of apoptotic processes could hinder the internalization of the dye. Notably, for I_spta_ = 15 mW/cm^2^ although this value is much lower than the cavitation threshold, cells are able to internalize 70 kDa dextrans, with a Stokes diameter of ~12 nm. This value can be taken as an estimate of the minimum size of the sonopores produced in the membrane. In Figure S6 (Section 4 of ESI) is reported a set of representative fluorescence images exhibiting the dextrans uptake at the threshold I_spta_ together with the PI test.

**Table 1:** Threshold I_spta_ values for the activation of the SP process for different MWs of the internalized dextrans, and their correspondent sonication doses.

Dextran MW (kDa)	Uptake threshold I_spta_ (mW/cm^2^)	Sonication dose (J/cm^2^)
10	9 ± 1	8.1 ± 0.9
40	12 ± 1	10.8 ± 0.9
70	15 ± 1	13.5 ± 0.9

The internalization of PI points out a significant cytotoxic response at I_spta_ = 15 mW/cm^2^ (exposure dose 13.5 J/cm^2^). As reported by Udroiu *et al*.^13^ in similar experimental conditions a slight but significant increase in the incidence of micronuclei was also observed in NIH-3T3 cells, depending on intensity and time of exposure to 1 MHz US. These observations suggested that further studies were required to better understand the bioeffects accompanying membrane SP, and therefore a study of the biological response of sonicated cells as a function of the US intensity was carried out.

**Figure 6:**
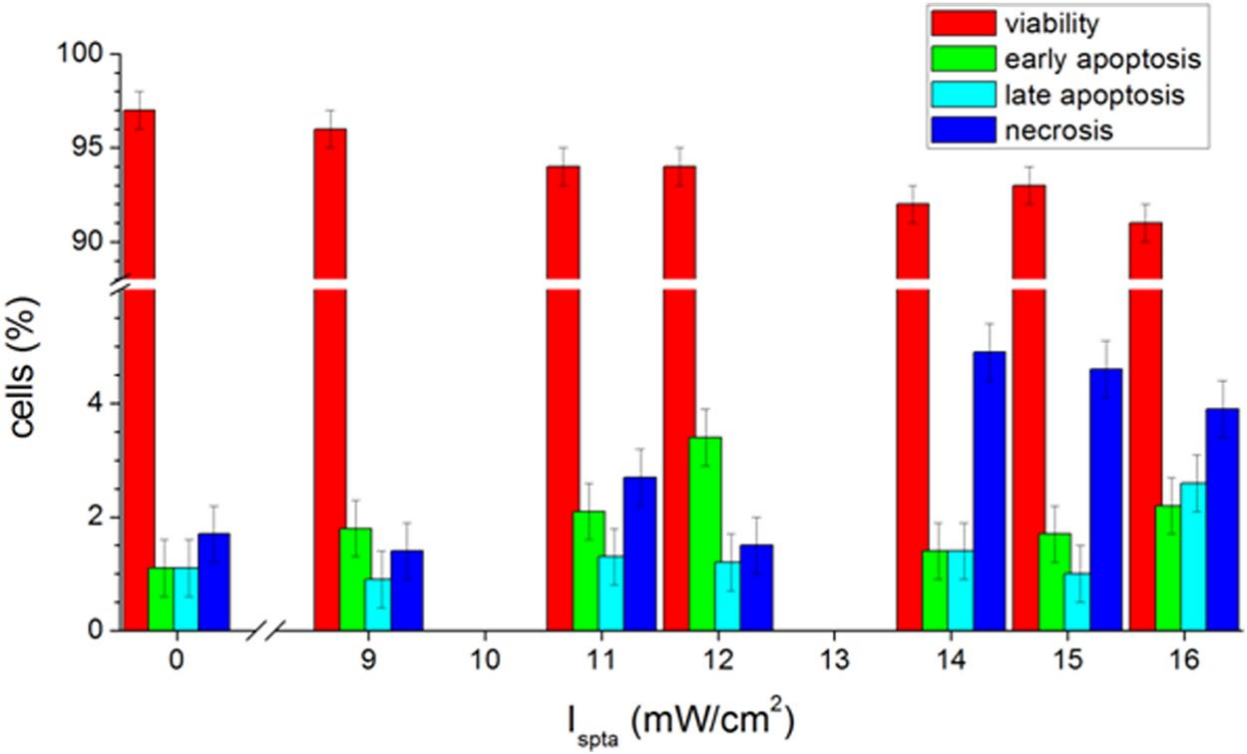
Viability and apoptosis vs necrosis evaluated by flow cytometry using a combined AnnexinV and PI assay on HaCaT cells sonicated for 15′ at 1 MHz, for different values of the I_spta_.

**Figure 7:**
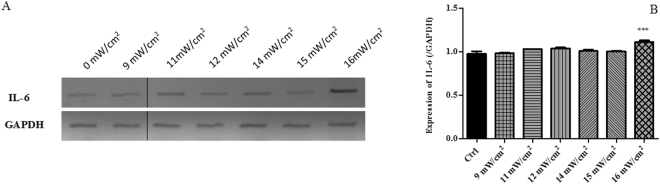
GAPDH and IL-6 mRNA levels analysed by RT-PCR (**A**) and Densitometric quantification (**B**). Columns and bars represent average and standard deviation value for three independent experiments. ****p ≤ 0.0001, ***p ≤ 0.001, **p ≤ 0.01, *p ≤ 0.05; all results were analysed by ANOVA, and the significance was evaluated by the Tukey honestly significant difference (HSD) post hoc test. All graphs were elaborated with Graph Pad Prism 6.0. Full length gels are presented in Section 6, Figure S8 of ESI. The gels were run under the same experimental conditions.

The biological response to the SP-induced “membrane wounds” induced by very low US intensities was thoroughly characterised by evaluating the cell death, using the combined AnnexinV and PI assay described in Section 2.5 (see also Section 5 of ESI). We also evaluated the expression of the gene IL-6, a cytokine linking chronic inflammation to cancer^33,34^. Results from the flow cytometry analysis, expressed in terms of cell percentage, are reported in Fig. 6. The cell viability is mainly preserved (>90%) during 15′ treatments, nevertheless, starting from I_spta_ = 9 mW/cm^2^ a slight decrease occurs, accompanied by an increase of the early apoptotic cells. At I_spta_ = 14 mW/cm^2^ a significant percentage of necrotic cells begins to be observed, while at I_spta_ = 16 mW/cm^2^ also late apoptosis contributes to cell death, in correspondence of a slight reduction of necrosis (representative flow cytometry dots plots in Figure S7 of ESI). IL-6 gene expression was determined by RT-PCR (Fig. 7). No up-regulation was observed for I_spta_ ≤15 mW/cm^2^ while the treatment at 16 mW/cm^2^ induced an overexpression of this gene compared to that of untreated cells.

These findings suggest that, starting from an I_spta_ threshold of 16 mW/cm^2^ and 15′ of sonication, the membrane damage caused by US not only decreases cell viability but also, through the overexpression of the inflammation-related gene IL-6, could induce an inflammatory response. Since several researches have recognized a role of the inflammation in promoting the pathogenesis and the progression of diseases such as cancer^35,36^, further investigation is needed to better characterise the aspects related to the inflammatory patterns.

### Analysis of the influence of the sonication dose

To quantitatively analyse the relation between the applied US field and the observed biological effects in terms of the energy delivered to the cells during the exposure, the sonication dose was calculated for each treatment as the product of the intensity I_spta_ by the exposure time. The obtained values are reported in Table 2, where the observed biological effects are identified by background colours. Our results suggest that the membrane SP occurs starting from a threshold dose of about 6.3 J/cm^2^. Moreover, the size of the sonopores can be related to the sonication dose. For doses higher than 10.8 J/cm^2^ a decrease of viability and concomitant events of early apoptosis are observed in the treated cell, followed starting from 14.4 J/cm^2^ by late apoptosis, necrosis, and overexpression of IL-6.

**Table 2:**
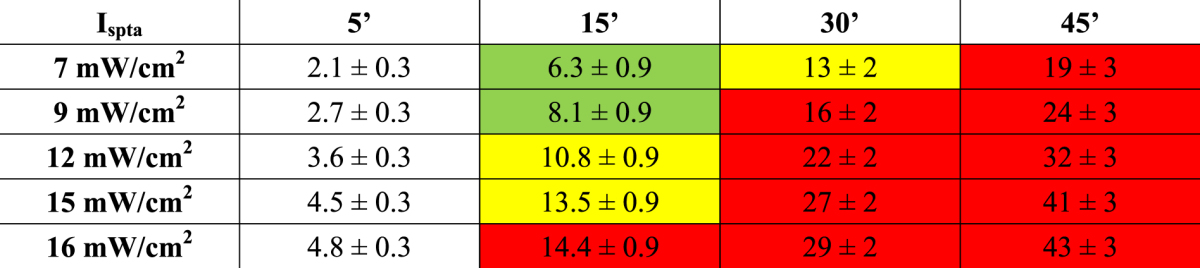
Sonication dose values calculated with respect to the treatments carried out on HaCaT cells and expressed in J/cm^2^; background colours correspond to the observed biological effects: no effects (white), membrane sonoporation (green), sonoporation + loss of viability (yellow), sonoporation + loss of viability including significant necrosis and apoptosis + inflammation (red).

We would stress that this dose, and I_spta_ 16 mW/cm^2^, corresponds to a negative peak pressure of ~0.02 MPa. This value seems high enough to induce mechanical stress at the plasma membrane/water interface, although it falls one order of magnitude lower than the safety exposure threshold value suggested so far (mechanical index ~0.2–0.3, at 1 MHz).

Notice that our experiments were compared successfully with those performed by using an electromedical system actually employed in US therapy (see also Section 2.2), thus highlighting the medical relevance of our approach. In this respect, we are confident that providing information on the sonication dose (at the fixed US frequency) allows a more complete index of characterization of the cell response to US exposure and, in turn, a fine identification of a range of parameters where transient SP occurs with negligible biological damages.

A very important step towards understanding the *in vivo* biological impact of our results would be to operate US exposures on three dimensional biosystems, such as human tissues composed by multi-stacked cheratinocytes or by blood brain barrier endothelial cells.

## Conclusions

*In vitro* effects of very low intensity (I_spta_~10 mW/cm^2^) 1 MHz US were studied on HaCaT cells using a versatile and well-calibrated homemade setup allowing for varying biomedical relevant exposure parameters (time, I_spta_) in a reproducible and effective manner. We focused on the possible US-induced alterations in the biological membrane permeability, cytotoxicity and the inflammatory gene response.

Specifically fluorescence microscopy and flow cytometry combined analyses shed new light on the possibility to trigger reparable sonoporation, well-below the cavitation regimes, to favour the uptake of membrane-impermeable drug-model molecules. Our findings disclosed a clear dependence of the uptake efficiency on the size of the internalized molecule through sonoporation, depending on both time and intensity of sonication. It is comforting that using the same setup conditions similar effects were obtained on murine fibroblasts NIH-3T3.

In this frame, we defined the sonication dose as the product of the I_spta_ by the sonication time which allowed us to identify a range of the exposure energy density (approximately between 6.3 J/cm^2^ and 10.8 J/cm^2^), useful to modulate membrane trafficking without relevant biological damages. However, by increasing the dose of exposure such an alteration was accompanied by a decline in cell viability and the activation of apoptosis. Moreover, we detected an overexpression of the IL-6 for sonication doses higher than ~15 J/cm^2^, and this is an object of future investigation. Our results might deserve some biomedical relevance, in a safe drug delivery scenario, for opening the protective barriers of many biosystems.

## Electronic supplementary material

Supplementary Information

## References

[1] Lindner JR (2004). Microbubbles in medical imaging: current applications and future directions. Nature Rev. Drug Discov..

[2] Oddo L (2017). Next generation ultrasound platforms for theranostics. J. Colloid. Interf. Sci..

[3] Capece S (2016). Complex interfaces in “phase-change” contrast agents. Phys. Chem. Chem. Phys..

[4] Mitragotri S (2005). Healing sound: the use of ultrasound in drug delivery and other therapeutic applications. Nat. Rev. Drug. Discov..

[5] Newman CM, Bettinger T (2007). Gene therapy progress and prospects: ultrasound for gene transfer. Gene Ther..

[6] Timbie KF, Mead BP, Price RJ (2015). Drug and gene delivery across the blood–brain barrier with focused ultrasound. J. Controll. Release.

[7] Zhou Y (2013). Ultrasound-mediated drug/gene delivery in solid tumor treatment. J. Healthc. Eng..

[8] Sivakumar M (2005). Transdermal drug delivery using ultrasound-theory, understanding and critical analysis. Cell. Mol. Biol..

[9] Liang HD, Tang J, Halliwell M (2010). Sonoporation, drug delivery, and gene therapy. Proc. Inst. Mech. Eng. H..

[10] Ter Haar G (2008). The resurgence of therapeutic ultrasound – a 21st century phenomenon. Ultrasonics.

[11] Tachibana K (2008). Sonodynamic therapy. Ultrasonics.

[12] Mason TJ (2011). Therapeutic ultrasound an overview. Ultrason. Sonochem..

[13] Udroiu I (2014). Potential genotoxic effects of low-intensity ultrasound on fibroblasts, evaluated with the cytokinesis-block micronucleus assay. Mutat. Res. Genet. Toxicol. Environ. Mutagen..

[14] Oberli MA, Schoellhammer CM, Langer R (2014). Ultrasound-enhanced transdermal delivery: recent advances and future challenges. Ther. Deliv..

[15] Park D (2016). Sonophoresis using ultrasound contrast agents: dependence on concentration. PLoS ONE.

[16] Konofagoua EE (2012). Ultrasound-induced Blood-Brain Barrier opening. Curr. Pharm. Biotechnol..

[17] Chu P-C (2016). Focused ultrasound-induced Blood-Brain Barrier opening: association with mechanical index and cavitation index analyzed by Dynamic Contrast-Enhanced Magnetic-Resonance Imaging. Sci. Rep..

[18] Niki E, Yamamoto Y, Komuro E, Sato K (1991). Membrane damage due to lipid oxidation. Am. J. Clin. Nutr..

[19] Dalecki D (2004). Mechanical bioeffects of ultrasound. Annu. Rev. Biomed. Eng..

[20] O’Brien WD (2007). Ultrasound-biophysics mechanisms. Prog. Biophys. Mol. Biol..

[21] Schlicher RK (2006). Mechanism of intracellular delivery by acoustic cavitation. Ultrasound Med. Biol..

[22] Domenici F (2013). Ultrasound well below the intensity threshold of cavitation can promote efficient uptake of small drug model molecules in fibroblast cells. Drug Delivery.

[23] Domenici F (2014). Structural and permeability sensitivity of cells to low intensity ultrasound: Infrared and fluorescence evidence *in vitro*. Ultrasonics.

[24] Krasovitski B, Frenkel V, Shoham S, Kimmel E (2011). Intramembrane cavitation as a unifying mechanism for ultrasound-induced bioeffects. P. Natl. Acad. Sci. USA.

[25] Atretkhany KN, Drutskaya MS, Nedospasov SA, Grivennikov SI, Kuprash DV (2016). Chemokines, cytokines and exosomes help tumors to shape inflammatory microenvironment. Pharmacol. Ther..

[26] Chungy J-I, Baruay S, Choiz BH, Min B-H (2012). Anti-inflammatory effect of low intensity ultrasound (LIUS) on complete Freund’adjuvant-induced arthritis synovium. Osteoarthritis Cartilage.

[27] Leskinen JJ, Hynynen K (2012). Study of factors affecting the magnitude and nature of ultrasound exposure with *in vitro set-ups*. Ultrasound in Med. & Biol..

[28] Guzmán HR, Nguyen DX, McNamara AJ, Prausnitz MR (2002). Equilibrium loading cells with macromolecules by ultrasound: effect of molecular size and acoustic energy. J. Pharm. Sci..

[29] Kodama T, Hamblin MR, Doukas AG (2000). Cytoplasmic molecular delivery with shock waves: importance of impulse. Biophys. J..

[30] Meijering BD (2009). Ultrasound and microbubble-targeted delivery of macromolecules is regulated by induction of endocytosis and pore formation. Circ Res..

[31] Sprawls, P. *Physical principles of medical imaging*, second ed. (Medical Physics Publishing Corporation, 1995).

[32] Moumarisa M, Rajoelyaa B, Abuaf N (2015). Fluorescein isothiocyanate-dextran can track apoptosis and necrosis induced by heat shock of peripheral blood mononuclear cells and HeLa cells. Open Biological Sciences Journal.

[33] Hunter CA, Jones SA (2015). IL-6 as a keystone cytokine in health and disease. Nat. Immunol..

[34] Fisher DT, Appenheimer MM, Evans SS (2014). The two faces of IL-6 in the tumor microenvironment. Semin. Immunol..

[35] Lin G, Reed-Maldonado AB, Lin M (2016). Effects and Mechanisms of Low-Intensity Pulsed Ultrasound for chronic prostatitis and chronic pelvic pain syndrome. Int. J. Mol. Sci..

[36] Silvestri I (2013). Effect of *Serenoa repens* (Permixon®) on the expression of inflammation-related genes: analysis in primary cell cultures of human prostate carcinoma. J. Inflamm..

