# Supplementary material for: Differential effects on membrane permeability and viability of human keratinocyte cells undergoing very low intensity megasonic fields
Source: Sci Rep. 2017 Nov 28;7:16536. doi: 10.1038/s41598-017-16708-4 (PMC5705699; doi:10.1038/s41598-017-16708-4)
Supplement: Supplementary file 1 — Supplementary Information [file 41598_2017_16708_MOESM1_ESM.pdf]

Differential effects on membrane permeability and viability of human keratinocyte cells undergoing very low intensity megasonic fields

ELECTRONIC SUPPORTING INFORMATION

F. Domenici[1*], F. Brasili[2], S. Giantulli[2], B. Cerroni[1], A. Bedini[3], C. Giliberti[3], R. Palomba[3], I. Silvestri[4], S. Morrone[5], G. Paradossi[1], M. Mattei[6], F. Bordi[2,7]

[1] Dipartimento di Scienze e Tecnologie Chimiche, Università degli Studi di Roma "Tor Vergata", Rome, Italy

[2] Dipartimento di Fisica, Università degli Studi di Roma "Sapienza", Rome, Italy

[3] Dipartimento Innovazioni Tecnologiche e Sicurezza degli Impianti, Prodotti e Insediamenti Antropici (DIT), INAIL, Monteporzio Catone, Rome, Italy

[4] Dipartimento di Medicina Molecolare, Università degli Studi di Roma "Sapienza", Rome, Italy

[5] Dipartimento di Medicina Sperimentale, Università degli Studi di Roma "Sapienza", Rome, Italy

[6] Centro Servizi Interdipartimentale - Stazione Tecnologia Animale and Dipartimento di Biologia, Università degli Studi di Roma “Tor Vergata”, Rome, Italy

[7] Istituto dei Sistemi Complessi, Consiglio Nazionale delle Ricerche, Florence, Italy

* Corresponding Author; e-mail: fabio.domenici@uniroma2.it

1. Ultrasound exposure system

The ultrasound (US) exposure device consists of a signal generator (Agilent 33220A), a signal amplifier (Amplifier Research 25A250) and a submersible piezo-ceramic circular transducer (44 mm diameter) tuned at 1 MHz (Precision Acoustics Ltd). The system can produce well defined sinusoidal US waves independently from the exposure conditions and allows to generate signals with different intensities, both in continuous and pulsed mode. A scheme of the exposure setup is reported below in Figure S1. It consists of a tank (30 × 30 × 30 cm) filled with degassed water, on whose bottom is placed the transducer. To isolate the effects of progressive waves, with respect to the reflected ones, the tank was coated with acoustic absorbers (Aptflex F28, Precision Acoustics Ltd). The temperature of the water bath was kept constant at 25°C. A hermetically lidded cell culture-treated Petri dish (9.6 cm^2) containing 3 ml of PBS solution was positioned at the water surface, submerged up to half of its thickness in line with the transducer.

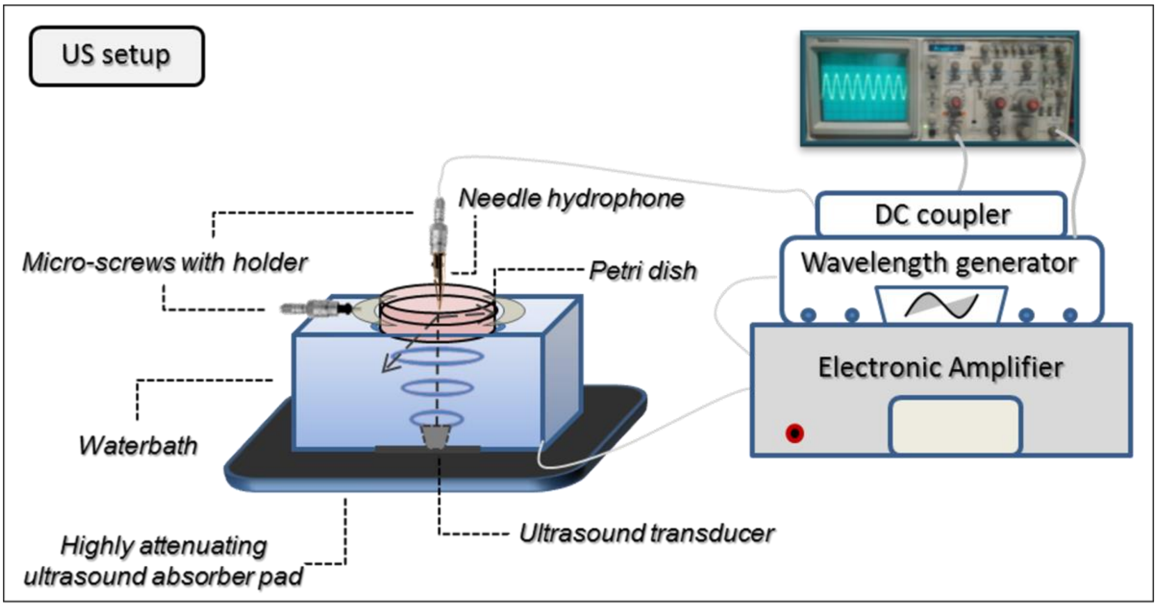

Figure S1. The ultrasonic exposure equipment consists of a versatile waveform generator, signal amplifier, and piezo-ceramic transducers. All parameters characterizing the transducer performance were measured by a needle hydrophone positioned within Petri dish, a booster amplifier (DC Coupler with Integral Power Supply, Precision Acoustics Ltd), and an oscilloscope. Maps of ultrasonic field were derived scanning a temperature controlled water tank with micrometric step-size movements. US absorber pads were used to avoid interferences from the reflected waves at the wall/water interface. Moreover the temperature inside and out the Petri dish was monitored through a digital thermometer.

2. Phase contrast and fluorescence microscopy (calcein uptake)

Images were acquired using an inverted, phase contrast fluorescence microscope Leica DMIL, endowed with a 100 W mercury vapour lamp and FITC filter set. The microscope is coupled with high resolution and sensitivity CCD photocam (Zeiss AxioCam ICc3) driven by Zen 2011 software. Petri dishes containing HaCaT cells (~70% confluent) were exposed to 1 MHz US at SSD of 7 cm, in presence 10 µM of fluorescent probe calcein. In Figure S2 below are shown representative images of samples treated with I_{spta} = (7 ± 1) mW/cm^2, at varying the exposure time in presence of calcein.

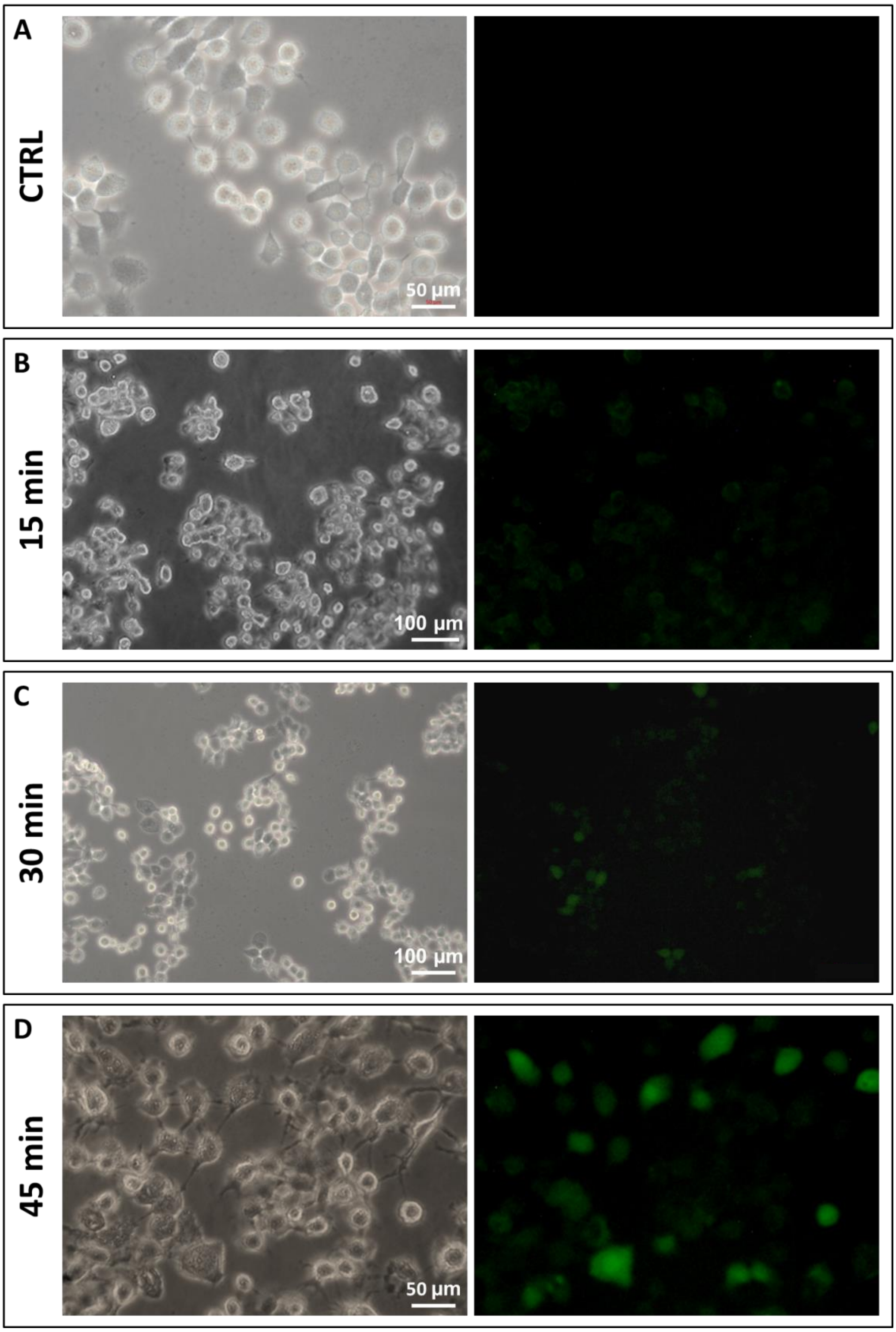

Figure S2. HaCaT cells sonicated with 1 MHz US at I_{spta} = 7 mW/cm^2, representative images of the control untreated sample (A) and of the samples treated for 15 min (B), 30 min (C) and 45 min (D) are shown. Phase contrast images of the sample (on the left) are compared with the corresponding FITC fluorescence images (on the right), where the uptake of the green dye calcein can be recognized.

3. Confocal laser scanning microscopy

Images were acquired using the Nikon Eclipse (Ti-E) inverted C1 confocal microscope, equipped with Ar ion (Spectra Physics, Mountain View, California) λ=488 nm and He-Ne (Melles Griot Florence, Italy) λ=543 nm lasers. All the sample was easily monitored with a motorized stage and the Nikon EZ-C1 software was used for acquisition. In Figure S3 below are shown images of samples treated with I_{spta} = 7 mW/cm^2 in presence of calcein, for different exposure times, while in

Figure S4 are reported different regions of interest of the sample after 30’ treatment. In Figure S5 a corresponding image showing quite rare cytotoxic events.

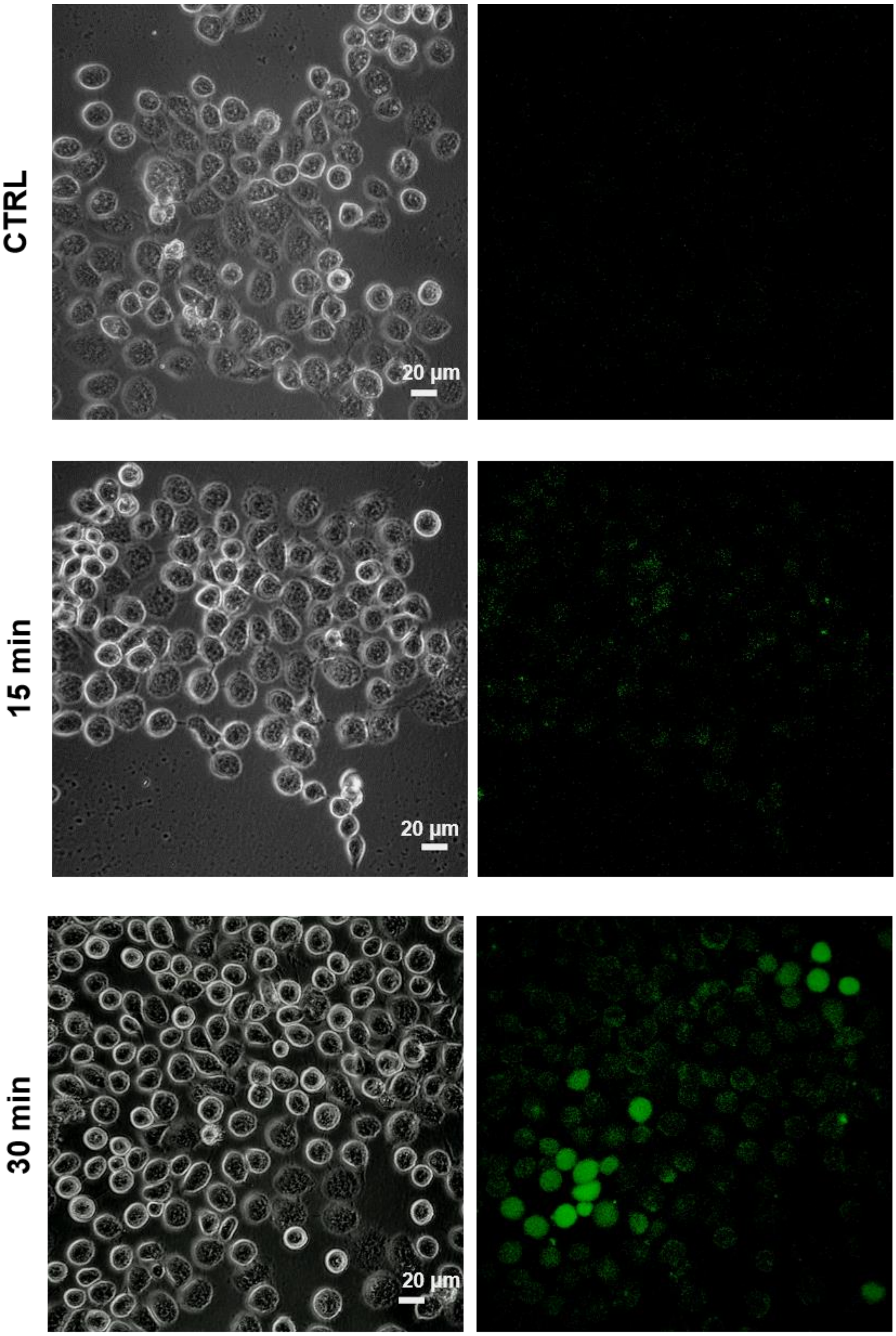

Figure S3. Phase contrast (on the left) and confocal laser fluorescence (on the right) images acquired on HaCaT cells treated for different exposure times with 1 MHz US at I_{spta} = 7 mW/cm^2. The different uptake of the green dye calcein at varying exposure time is shown.

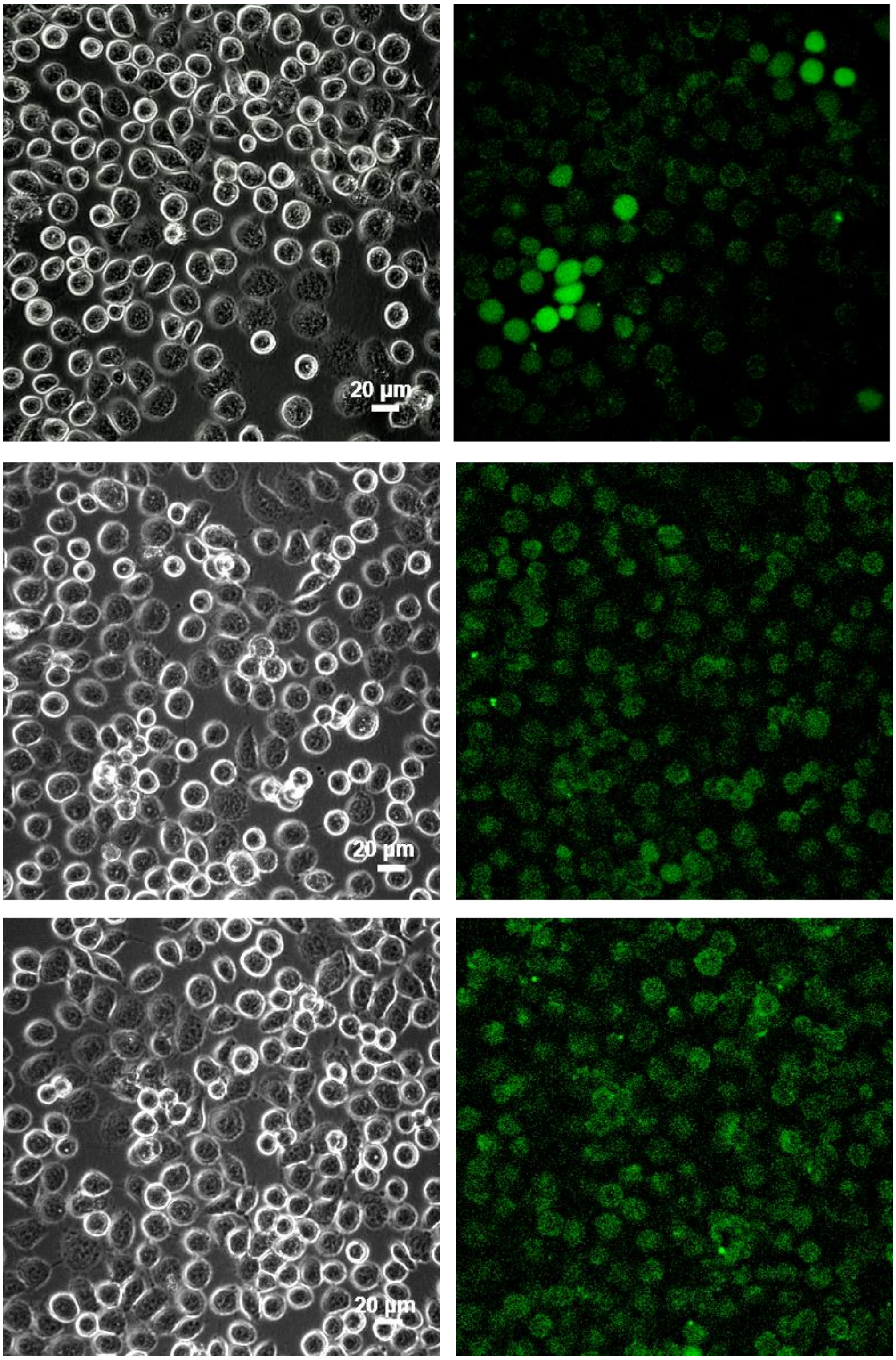

Figure S4. Phase contrast (on the left) and confocal laser fluorescence (on the right) images acquired on different regions of the HaCaT cell sample treated for 30’ with 1 MHz US at I_{spta} = 7 mW/cm^2.

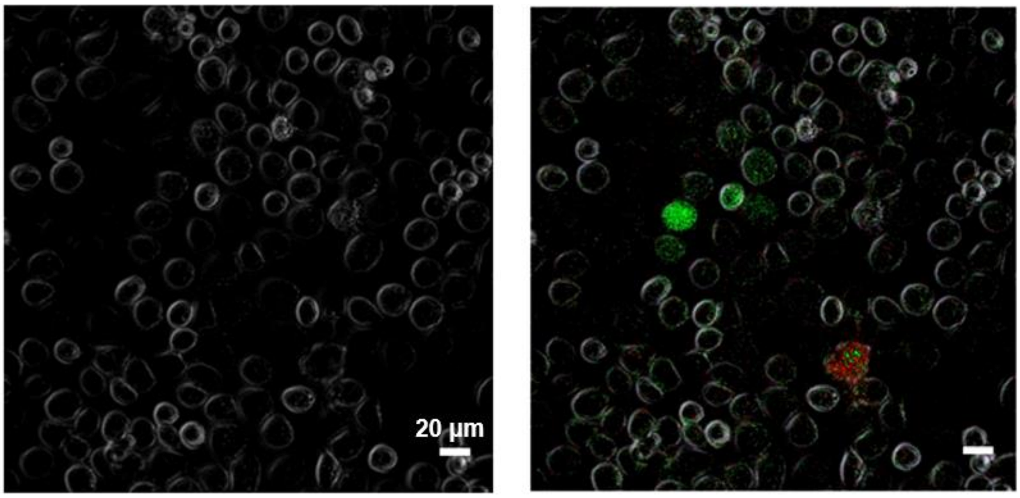

Figure S5. Phase contrast (on the left) and confocal laser fluorescence (on the right) images of the HaCaT cells sample treated for 30’ with 1 MHz US at I_{spta} = 7 mW/cm^2. Here, one of the few cases of the colocalization of the two dyes (calcein and PI) is detected.

4. Phase contrast and fluorescence microscopy (dextrans uptake with PI assay)

Petri dishes containing HaCaT cells (~70% confluent) were exposed to 1 MHz US at SSD of 7 cm, in presence 10 µM of FITC labelled dextrans with different MWs. In Figure S6 are shown representative phase contrast and fluorescence microscopy images of samples sonicated for 15’, at the I_{spta} needed for the dextrans to enter the cell, with the corresponding cytotoxic assay by PI probe.

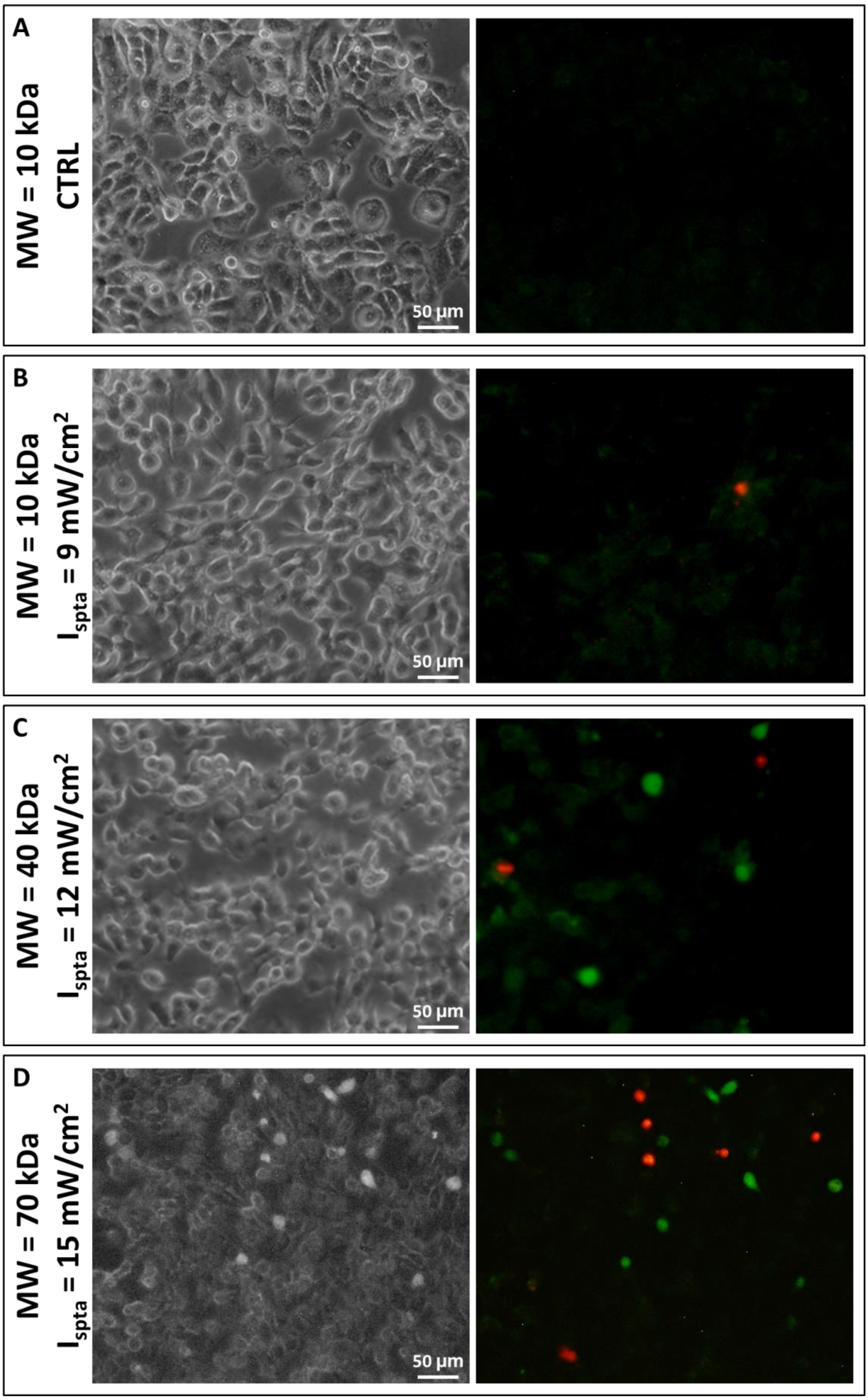

Figure S6. HaCaT cells sonicated with 1 MHz US for 15' in presence of FITC labelled dextrans with different MWs. Representative phase contrast images of the control untreated sample (A) and of the samples treated with the threshold I_{spta} corresponding to each MW: 9 mW/cm^2 for 10 kDa (B), 12 mW/cm^2 for 40 kDa (C) and 15 mW/cm^2 for 70 kDa (D). In the corresponding fluorescence images shown on the right, the dextran (green) and the PI (red) uptake point out the membrane SP and the cytotoxic response, respectively.

5. Cytotoxicity at increasing I_{spta} by FACS

AnnexinV and PI combined assay was performed in triplicate as follows. After the treatment the cells were dispersed in Dulbecco's Phosphate-Buffered Saline and transferred in Falcon tubes for centrifugation. The pellet was then suspended in 1 ml binding buffer volume (10 mM Hepes/NaOH, pH 7.4, 140 mM NaCl, 2.5 mM $CaCl_2$; filtered through 0.2 µm sterile pore filter), bearing in mind that the cell density should fall in the range of 2-5 × 10^5 cells/ml for reading by FACS. 195 µl of cell suspension were mixed with 5 µl of AnnexinV-FITC (solution containing 50 mM TRIS, 100 mM NaCl, 1% BSA, 0.02% Sodium Azide, pH 7.4), and incubated for 10' in the dark. Immediately after incubation the dispersion was dispersed in Hepes (final volume of 2 ml) and centrifuged. The cells were resuspended in 400 µl of binding buffer and just before FACS reading we have put 10 µl of PI stock solution (100 µg/ml in PBS).

Most significant FACS dots plots are shown in Figure S7, as representative of control sample and 15' treatments at I_{spta} = 9 mW/cm^2 (some early apoptotic cells), I_{spta} = 14 mW/cm^2 (some necrotic cells), and I_{spta} = 16 mW/cm^2 (significant necrotic cells and some late apoptosis).

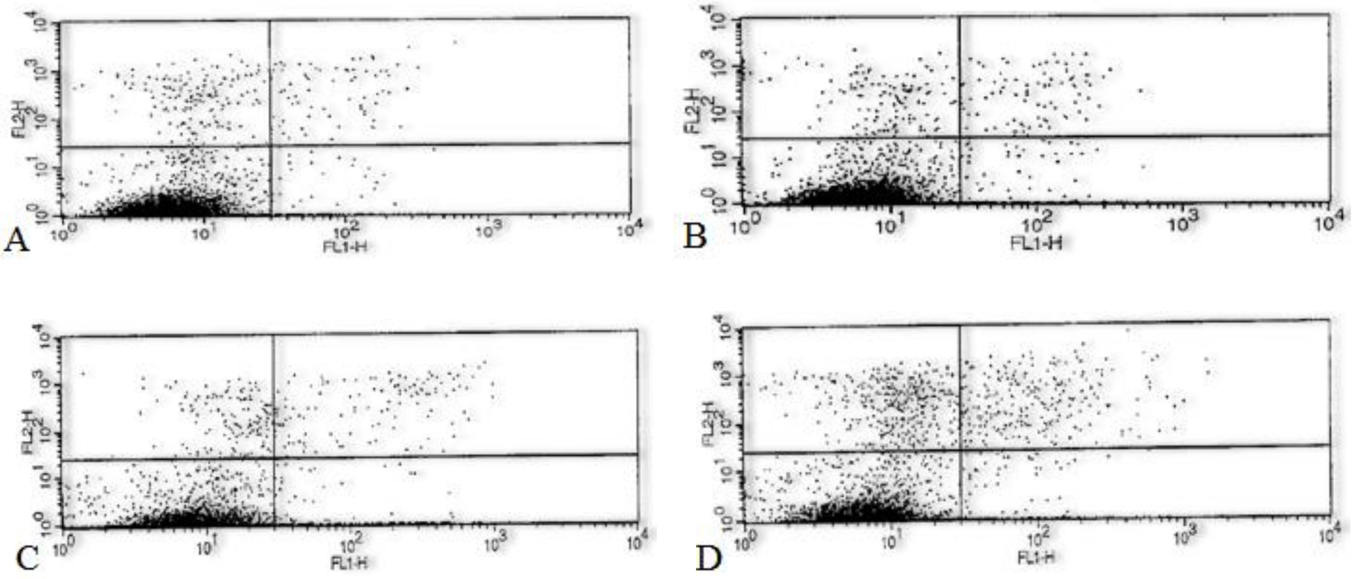

Figure S7. FACS dot plots (FL1, Annexin; FL2, PI), representative of untreated samples (A), and of 1 MHz US treatments for 15' at I_{spta} = 9 mW/cm^2 (B), I_{spta} = 14 mW/cm^2 (C), and I_{spta} = 16 mW/cm^2 (D).

6. RT-PCR

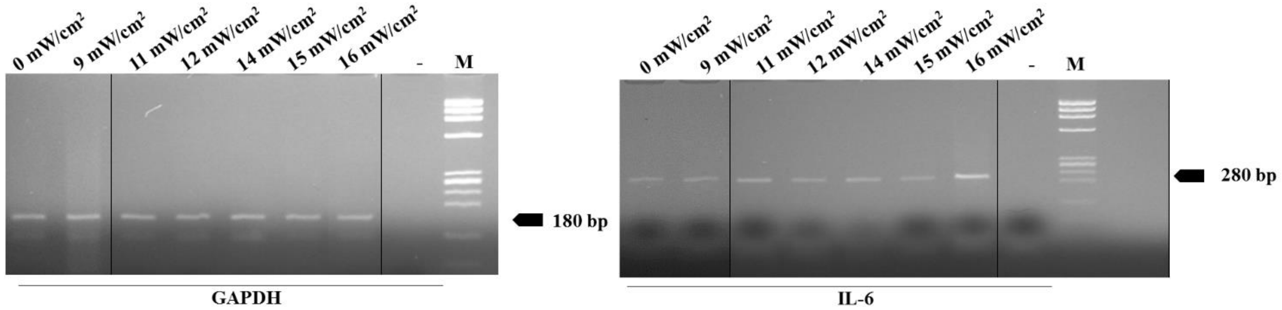

Figure S8. Full-length gel results of Figure 7A. RT-PCR shows the expression level of GAPDH (left) and IL-6 (right) in HaCaT cell line. The thermal profile for PCR amplification was as follows: GAPDH, 94 °C for 30", 60 °C for 30", 72 °C for 30" for a total of 30 cycles; IL-6 (right), 95 °C for 50", 56 °C for 1', 73 °C for 15" for a total of 30 cycles.
